# De-medicalization of birth by reducing the use of oxytocin for augmentation among first-time mothers – a prospective intervention study

**DOI:** 10.1186/s12884-018-1706-4

**Published:** 2018-03-27

**Authors:** L. C. Gaudernack, K. F. Frøslie, T. M. Michelsen, N. Voldner, M. Lukasse

**Affiliations:** 1 Department of Obstetrics and Gynecology, Rikshospitalet, Oslo University Hospital / Oslo and Akershus University College of Applied Sciences, Oslo, Norway; 2 Norwegian National Advisory Unit on Women’s Health, Oslo, Norway; 3 Department of Obstetrics and Gynecology Rikshospitalet and National Advisory Unit on Women’s Health, Oslo University Hospital, Oslo, Norway; 4 Faculty of Health Studies, VID Scientific International Diaconal Specialized University Oslo, Oslo, Norway; 5 Faculty of Health Sciences, Oslo and Akershus University College of Applied Sciences, Oslo, Norway

**Keywords:** First-time mothers, De-medicalization, Oxytocin augmentation, Operative vaginal delivery, Labor duration

## Abstract

**Background:**

The use of synthetic oxytocin for augmentation of labor is rapidly increasing worldwide. Hyper-stimulation is the most significant side effect, which may cause fetal distress and operative delivery. We performed an intervention consisting of an educational program and modified guidelines to achieve a more appropriate use of oxytocin.

**Methods:**

This prospective intervention study included 431 first-time mothers at term with spontaneous onset of labor before (October 2012 to May 2013), and 664 after the intervention (April 2014 to April 2015). Our outcomes were prevalence and duration of oxytocin treatment, mode of delivery, indication for operative delivery, episiotomy, anal sphincter tears, bleeding, labor duration, pain relief and the effect of oxytocin on mode of delivery.

**Results:**

After the intervention, 52.9% were diagnosed with dystocia, compared with 68.9% before (*p* < 0.001). Oxytocin was not always used in accordance with the guidelines, but a significant reduction in oxytocin rates from 63.3% to 54.1% (*p* < 0.001) was obtained. More women without dystocia according to the existing guidelines were augmented after the intervention (18.9% vs 8.4%, *p* < 0.001). Assessing all labors, the median duration of oxytocin treatment was reduced by 72% (from 90 to 25 min) without increasing the median duration of labor (385 min in both groups). There was a moderate reduction in operative vaginal deliveries from 26.9 to 21.5% (*p* = 0.04), and dystocia as an indication for these deliveries increased (*p* = 0.01). There was a moderate increase in caesarean sections from 6.7 to 10.2% (*p* = 0.05), but no increase in dystocia as an indication for these deliveries. Women receiving oxytocin were more likely to have an operative vaginal birth, even after adjusting for birth weight, epidural analgesia and labor duration, OR: 2.1 (CI 1.1-4.0) before and OR 2.7 (CI 1.6-4.5) after the intervention.

**Conclusions:**

Our intervention led to a significant reduction in the use of oxytocin. However, more than half of the women remained diagnosed with dystocia. Operative vaginal births seem to be associated with oxytocin treatment. Therefore, augmentation with oxytocin should be used with caution and only when medically indicated. Even more modified guidelines for augmentation than the ones applied in this study might be appropriate.

## Background

Augmentation of labor with synthetic oxytocin is increasingly used worldwide [1–4]. Its purpose is to avoid or treat dystocia (prolonged labor) and associated adverse outcomes, such as a negative birth experience and cesarean section on maternal request in next pregnancy [5, 6], chorioamnionitis, operative birth and post-partum hemorrhage [7–9]. The definition, incidence and treatment of dystocia vary both nationally and internationally. The World Health Organization (WHO) defines dystocia as 4 h without progress in the active phase of labor (https://www.ncbi.nlm.nih.gov/books/NBK258881/). In contrast, according to the concept “Active management of labor”, dystocia is defined as a dilatation rate of less than 1 cm per hour [10].

Oxytocin has adverse effects. Excessive use of the drug leads to uterine hyper-stimulation, which impairs oxygen supply to the fetus and can result in fetal asphyxia and, in severe cases, can cause permanent impairment [11–13]. Continuous fetal monitoring is indicated when augmenting labor. The monitoring can prevent women from using movement, massage and water to ease labor pain and may therefore lead to increased use of epidural analgesia for pain relief. Several studies have demonstrated correlations between the use of oxytocin, operative birth, low Apgar scores and transfer to neonatal intensive care units [1, 9, 12, 13]. The risk of operative birth is even more elevated when labor is augmented without existing dystocia [1, 12]. A significant association has been shown between augmentation with oxytocin in first labor and anal sphincter injuries [14], post-partum urinary retention [15], postpartum hemorrhage [16] and delayed initiation of breastfeeding [17]. According to findings from the Norwegian Mother-Child cohort study, 70% of first-time mothers want a birth without medication, yet only 29% of these women experience such a labor [18].

Recent studies report an increasing and random use of oxytocin, especially among first-time mothers, [1, 2, 9, 19]. In primiparous women with a single fetus, cephalic presentation and spontaneous onset of labor (Robson group 1) [20], the prevalence of augmentation is reported to be 37-75% [1, 2, 9, 10, 12]. Some studies report that 40-50% of the women treated with oxytocin did not have dystocia [1, 2].

We initiated an intervention study aiming to achieve a more appropriate use of oxytocin. The intervention combined a change in the oxytocin guidelines with an educational program. We hypothesized that the intervention would result in a reduction in the overall use of oxytocin and that the drug to a larger extent would be given only to women with dystocia. We also hypothesized that a reduction in the use of oxytocin would take place without an increase in operative deliveries, or compromising obstetric and neonatal outcomes. We therefore investigated whether there was an association between oxytocin and the incidence of operative deliveries. In addition we examined the effect of the intervention on the proportion of women receiving oxytocin, duration of oxytocin treatment, duration of labor, prevalence of episiotomy, anal sphincter tears, pain relief, bleeding, mode of delivery, and indication for operative births.

## Methods

This intervention study occurred in a delivery ward at a Norwegian University Hospital. Data were collected in two periods: before the intervention, from October 2012 through May 2013, and after the intervention, from April 2014 to April 2015. The electronic programs used at our hospital ensure high quality data with few missing values. To follow the effect of the intervention, the collection period after the intervention was longer, as the effect of quality improvement interventions is known to diminish with time. All women in Robson group 1 were included, except for four women for whom data regarding dystocia were not available.

The study hospital had strict guidelines for dystocia. In the period before the intervention we used the principles of Proactive Labor Support [21] i.e. oxytocin was indicated when dilatation was slower than 1 cm per hour, and labor started at 1 cm, with an enfaced cervix and painful regular contractions. Using this definition, the vast majority of women in Robson group 1 were diagnosed with dystocia and augmented with oxytocin prior to the study intervention.

After merging between the study hospital and another hospital, new guidelines were adopted. In the new guidelines for use of oxytocin, the definition of labor start remained unchanged, but oxytocin indication was changed from less than 1 cm per hour to 2 h without progress. For the second stage of labor, guidelines were not changed; oxytocin was to be initiated after 1-2 h without initiation of pushing or if the pushing period lasted more than 1 h.

On diagnosis of dystocia, the first line of treatment was amniotomy. In case of no progress 1-2 h after amniotomy, oxytocin infusion was started. As part of the intervention, all of the midwives attended an educational program on the new guidelines and advantages and disadvantages of oxytocin. The project leader (LCG) held a lecture compulsory to all midwives and doctors at the maternity ward. Posters with the new guidelines were placed beside all computers and in all delivery rooms in the unit. To inform the staff of the results and to inspire further efforts to use oxytocin in agreement with the new guidelines, every 4-8 weeks, we displayed (SPC) Statistical Process Control charts to visualize the proportion of primiparous women receiving oxytocin.

We collected data from medical records. Duration of labor was calculated in minutes from the first vaginal examination on the partogram to birth. Documentation in the partogram was started once a woman was in established active labor. When presenting results of this study the term dystocia is defined as women having dystocia according to existing guidelines in the period they gave birth.

### Statistical analyses

Descriptive statistics are given as the means and standard deviations (SD), medians and quartiles (Q_1_, Q_3_) or frequencies and percentages (%), depending on the data distribution.

SPC (Statistical Process Control) was used to visualize and analyze the temporal trend in the proportion of women being treated with oxytocin before and after the intervention. We used a P-chart with months as the time unit. Briefly, a P-chart displays identically defined proportions in consecutive independent samples, at equally spaced time points. According to SPC theory, a significant change is observed when eight subsequent observations after an intervention are below the mean of the period before the intervention [22].

Comparisons of maternal and fetal factors, labor duration, oxytocin use and obstetric outcomes before and after the intervention were achieved using two sample t-tests, Mann-Whitney tests or Chi square tests, depending on the type and distribution of the data.

The effect of oxytocin on operative vaginal delivery was estimated by logistic regression analysis, with operative vaginal births versus spontaneous vaginal births as the outcome. The main exposure variable was oxytocin treatment (yes or no). Factors known to affect the risk of operative vaginal birth and treatment with oxytocin were included in multiple analyses, including birth weight, epidural analgesia and duration of labor, and in supplementary analyses BMI (Body mass index), maternal age and fetal presentation at birth. Due to few cesarean sections in subgroups, a possible association between oxytocin and caesarean sections was not possible to study.

All analyses were performed in SPSS (version 23). A *p*-value less than 0.05 was considered statistically significant.

In order to investigate differences in the proportion of primiparous women classified as Robson 1 (spontaneous labor start) or Robson 2a (induced labor start) before and after the intervention, we studied data from the labor ward’s electronic databases.

### Ethical considerations

Before the intervention was started, the Regional Committee for Health and Research Ethics (REC) was asked for permission. The REC considered the project to be a quality improvement project and stated that ethical approval was not necessary (REC number/Do. id: 206053/23. Aug. 2011). The Personal Data Officer (PVO) at our hospital approved the study and stated that informed consent was not necessary. Data were stored in a password-protected database, where only the project leader had access. All data were de-identified.

## Results

We included 431 women before and 664 women after the intervention. There were no significant differences in maternal or fetal characteristics between the groups of women before and after the intervention concerning pre-pregnancy BMI, age, marital status, gestational age, birthweight, 5-min Apgar score or duration of labor (Table 1).

**Table 1:** Maternal, fetal and obstetric characteristics before and after the intervention (mean (SD), median (Quartiles) or frequency (%) unless otherwise stated)

Study sample - all women in the study	Before the intervention *n* = 431	%	After the intervention *n* = 664	%	*p*-value
Maternal age	31.2 (4.6)		30.9 (4.5)		0.26
Co-habiting or married	412	95.6	627	94.4	0.39
BMI	22.55 (3.5)		22.66 (3.6)		0.63
Gestational age	281.8 (7.6)		281.2 (7.7)		0.22
Birth weight	3443 (460)		3444 (409)		0.95
5 min Apgar score > = 7	428	99.3	661	99.5	0.55
Duration of labor in minutes - median (Quartiles)	385 (233-550)		385 (230-609)		0.55
Duration of oxytocin in minutes - median (Quartiles)	90 (0-270)		25 (0-195)		< 0.001 (diff:65 min)
Oxytocin for augmentation	273	63.3	359	54.1	< 0.001
Characteristics of oxytocin use -all women receiving oxytocin			*n* = 359		
Oxytocin without dystocia	23	8.4	67	18.7	< 0.001
Oxytocin started at > = 6 cm dilatation	140	51.3	220	61.3	0.01
Oxytocin started in first stage of labor	210	76.9	232	64.6	< 0.001

**Fig. 1:**
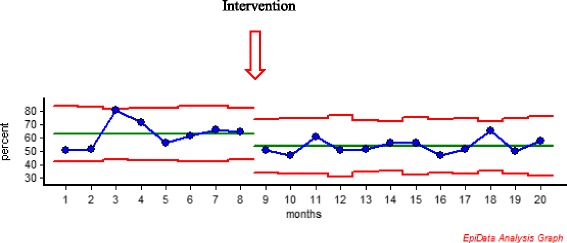
Use of oxytocin before and after the intervention the figure shows monthly proportion of women in Robson group 1 receiving oxytocin for augmentation before and after the intervention, which took place after month 8. The first nine registrations after the intervention are placed under the mean for the period before the intervention, According to SPC theory, a significant change is observed when eight subsequent observations after an intervention are below the mean of the period before the intervention

**Fig. 2:**
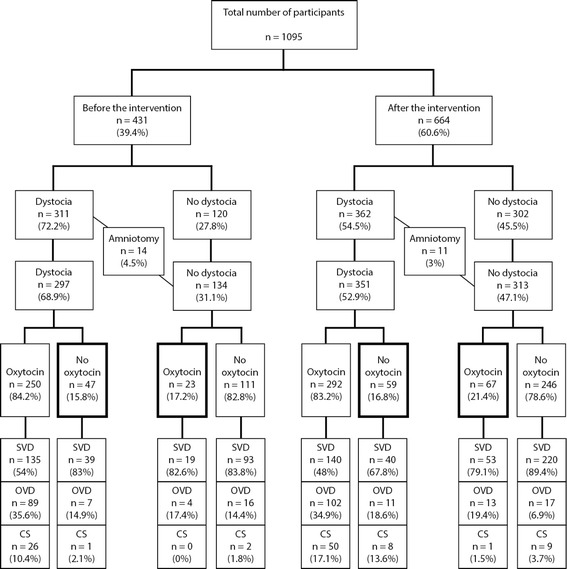
Augmentation with oxytocin, dystocia and mode of birth before and after the intervention. The ones in balled frames are those not treated correctly with oxytocin or no oxytocin, according to existing guidelines. SVD = spontaneous delivery, OVD = operative vaginal delivery, CS = caesarean section

Oxytocin was used in significantly fewer cases after the intervention (Fig. 1). In total, 63.3% were augmented with oxytocin before and 54.1% after the intervention (*p* < 0.001) (Figs 1 and 2). The intervention led to a highly significant reduction in the duration of oxytocin treatment, with 72% less time with oxytocin per woman after the intervention (90 vs 25 min), while median duration of labor was unchanged (385 min in both groups) (Table 1).

According to the guidelines used before the intervention, 68.9% of the women had dystocia. After the intervention, using the new guidelines, 52.9% had dystocia (Fig. 2). After the intervention, oxytocin was given in significantly more cases without dystocia (18.9% vs 8.4%), and in significantly fewer cases oxytocin was started in first stage of labor (76.9% vs 69.3%). There were also significantly fewer women being augmented before 6 cm of cervix-dilatation after the intervention (Table 1).

Significantly more women used water and received pudendal for pain relief after the intervention, and significantly fewer women had an amniotomy performed (Table 2). No significant changes were found in the prevalence rates of episiotomy, anal sphincter tears, use of epidural analgesia or amount of postpartum bleeding. There were only six newborns with 5 min Apgar scores less than 7, three before and three after the intervention.

**Table 2:** Obstetric outcomes and indications for operative delivery

	Before the intervention *n* = 431		After the intervention *n* = 664		
	n	%	n	%	*p*-value
Water as pain relief	105	24.4	224	33.7	0.001
Epidural analgesia	280	65.0	409	61.6	0.26
Pudendal analgesia	28	6.5	106	16	< 0.001
Amniotomy	206	47.8	275	41.4	0.04
Episiotomy	141	32.7	215	32.4	0.91
Third degree perineal tears	7	1.6	14	2.1	0.67
Mean blood loss in ml (SD)	435 (257)		462 (299)		0.11
Spontaneous vaginal birth	286	66.4	453	68.2	0.55
Operative vaginal delivery	116	26.9	143	21.5	0.04
Indications for Operative vaginal birth					
Dystocia	34	29.3	66	46.2	0.01
Asphyxia	61	52.6	69	48.2	0.53
Other reasons	21	18.1	8	5.6	0.002
Emergency caesarean section	29	6.7	68	10.2	0.05
Indications for CS					
Dystocia	17	58.6	34	50.0	0.51
Asphyxia	9	31	26	38.3	0.64
Other reasons	3	10.3	8	11.7	0.57

There was a significant difference in mode of delivery, as fewer women had an operative vaginal birth and more women had intrapartum cesarean section after the intervention. Significantly more operative vaginal births were performed due to dystocia after the intervention, and due to other reasons before the intervention. No differences were found in indications for cesarean sections (Table 2).

Among women not having dystocia after the intervention (*n* = 313, Fig. 2), we observed more operative vaginal births (19.4%, *n* = 13) among those treated with oxytocin than among those not being treated with oxytocin (6.9%, *n* = 17). However, the numbers in the subgroups were too small to perform regression analyses with adjustment for confounding variables.

When comparing mode of delivery in women augmented with oxytocin, with those not augmented, we found that oxytocin remained a significant risk factor for operative vaginal birth, both before and after the intervention, even after adjusting for birth weight, labor duration and epidural analgesia; OR 2.1, (CI 1.1-4.0) before and OR 2.7 (CI 1.6-4.5) after the intervention (Table 3). In supplementary analysis, we also adjusted for maternal age, BMI, and fetal presentation at birth, resulting in negligible changes in the OR for operative vaginal birth.

**Table 3:** OR for operative vaginal delivery in women augmented with oxytocin, compared to women not augmented, before and after the intervention. Women with caesarean sections are excluded

		OVD	SVD	OR/CI Unadjusted	*p*-value Unadjusted	OR/CI Adjusted ^b^	*p*-value Adjusted
Before the intervention *N* = 402	Oxytocin^a,b^*n* = 241	90 (34%)	151	3.1(1.9 -5.1)	< 0.001	2.1 (1.1-4.0)	0.026
	No oxytocin *n* = 161	26 (10%)	135		1 (ref)		
After the intervention *N* = 596	Oxytocin^a,b^*n* = 288	107 (32%)	181	4.7 (2.9 -6.8)	< 0.001	2.7 (1.6-4.5)	< 0.001
	No oxytocin *n* = 308	36 (11%)	272		1 (ref)		

CS: *n* = 29 before and *n* = 68 after the intervention are excluded

^a^ Oxytocin for more than 20 min

^b^ Adjusted for labor duration, birth weight and epidural

Response variables: *OVD* operative vaginal delivery, *SVD* spontaneous vaginal delivery

There were no differences in the proportion of primiparous women classified as Robson 1 (spontaneous labor start) or Robson 2a (induced labor start) before and after the intervention. Data from 2013 showed that 27.4% of primiparous women with one fetus in cephalic presentation had an induced labor and thus were in Robson group 2a. For the period after the intervention 24.6% of these primiparous women had an induced labor. This difference is not statistically significant.

## Discussion

As hypothesized, the intervention contributed to a significant reduction in the use of oxytocin for augmentation; fewer women received oxytocin, and the women received oxytocin for a shorter period. However, in contrast to the intention, oxytocin was used significantly more often despite no dystocia after the intervention. There was a moderate decrease in operative vaginal births and a moderate increase in cesarean sections after the intervention. Oxytocin treatment was a significant risk factor for operative vaginal birth both before and after the intervention.

All data after the intervention was collected prospectively, which is a strength of our study. The study was based on strict diagnostic criteria, as the definition of dystocia and the guidelines for when to use oxytocin were clear and concise. A better design would have been a randomized controlled trial comparing two different approaches in primiparous women diagnosed with dystocia. However, this design was not possible in our setting, as we used data already collected (before the intervention) in order to compare with data collected prospectively (after the intervention).

To our knowledge, comparisons of mode of delivery in women treated with oxytocin with those not treated have only been made in a few earlier studies [1, 5, 12]. Another strength in our study is the high detailed quality of our data on every labor, allowing adjustments for important confounding factors.

Our intervention could have been more efficient. The educational program should have been presented more than once. To facilitate appropriate use of oxytocin, we could have used checklists, as in the studies by Holmgren [23] and Clark [24]. We could also have used stake holders among the labor ward personnel, discussing and promoting the issue of proper use of oxytocin, as in the study by Holmgren et al. [23]. These stakeholders could have contributed to more awareness among the midwives regarding oxytocin and possibly a more correct use of the drug.

A possible explanation why more oxytocin was used in the absence of dystocia after the intervention, may be that it is hard to adjust to new guidelines. It might be a natural effect of a changed routine, as not all personnel remembered to use the new guidelines. Another reason could be a busy labor ward putting pressure on midwives to complete births quickly.

Advantages of the new guidelines included the significantly shorter duration of oxytocin treatment. According to our guidelines, fetal surveillance should always be performed when oxytocin is used to augment labor. Less time with oxytocin treatment therefore means less time attached to a CTG or STAN monitor. This might explain why the women used more water for pain relief after the intervention.

One to one care during the active stage of labor is an important measure to promote normal labor [25]. A change in the availability of this kind of care between the period before and after the intervention could have influenced on the normal birth rate. However, there was no change in the ward’s practice or guidelines. Both before and after the intervention, the guidelines emphasized that one to one care is important to keep birth normal.

The intervention in the present study, allowing 2 h instead of 1 h without progress before dystocia was diagnosed, resulted in fewer oxytocin treatments before 6 cm. This is in accordance with findings of two large studies on progress of first time labor by Zhang et al. [26] and Neal et al [27], who show that normal progression in labor accelerates only after 6 cm. Thus, it might be that the intervention resulted in a more appropriate use of oxytocin.

Our study showed a small but significant increase in cesarean sections. One could assume this increase was due to failure to progress, but there was no increase in dystocia as an indication for cesarean sections. The cesarean section rate in Robson group 1 was unusually high at the study hospital in the data collection period after the intervention (10.2% from 1.4.2014 to 1.4.2015). For the years 2015 and 2016, the cesarean rates in Robson group 1 were 7.1% and 6.6%, respectively (http://statistikkbank.fhi.no/mfr), despite no change in the guidelines for dystocia. It is likely that the increase in cesarean sections after the intervention was due to other causes than the change in the use of oxytocin.

The prevalence of dystocia in our study was high, even after the intervention, compared with two other cohort studies by Kjergaard et al. [9] and Bernitz et al. [1], These studies included a similar population of first-time mothers and similar guidelines for dystocia, as in our study after the intervention. Their dystocia rates were 37% [9] and 25.2% [1] compared with 52.9% in our study after the intervention. In contrast to these studies, we defined labor start to be at 1 cm, while they defined labor start at 4 cm. As labor is known to progress more slowly in the beginning of the first stage [26], more women were diagnosed with dystocia in our study, this in turn might have contributed to more operative vaginal deliveries. The definition of labor start used in our study, differs from WHO’s much used definition of 4 cm, which is also the most commonly used definition in Norway. Our definition was chosen to avoid long latent phases, which are associated with both maternal and fetal risks as caesarean section, low Apgar score and admission to Neonatal Care Unit [28], and negative birth experience [6, 29]. Further research is necessary to study whether women with long latent phases are more at risk for operative vaginal delivery, i.e. if they have to wait until cervix is 4 cm dilated before they can be treated with oxytocin, compared to if they receive this treatment earlier.

Both vaginal operative births and episiotomies should be used restrictively, as both are risk factors of negative birth experiences and maternal request for cesarean section [30]. Our findings suggest an increased risk for operative vaginal birth when being augmented with oxytocin both before and after the intervention. In the present study the rates of operative vaginal births were 26.9% before and 21.5% after the intervention. We are not aware of any changes taking place between the period before and of the intervention that could have influenced on the rate of operative vaginal deliveries. We know of no courses, conferences or projects encouraging or discouraging OVD.

The higher rate of operative vaginal births in our study compared to 14.6% in the study by Kjergaard et al. [9] and 16% in the study by Bernitz et al. [1], might be associated with our higher rate of augmentation. The explanation for this higher rate may partly be because the guidelines were still too strict, resulting in augmentation of some women who were not really in need of oxytocin. The two other studies had augmentation rates of 36.5% [9] and 43.9 [1] compared with 53.9% in the present study. In addition, 8.4% of the augmented women before and 18.9% after the intervention did not have dystocia. Furthermore, after full dilatation, the studies by Bernitz and Kjergaard allowed 2 h before active pushing and 3 h for women with an epidural, instead of 1-2 h for all women, as in the our study. In our study, in many cases, oxytocin was started 1 h after full dilatation. It is likely that pushing was initiated too early in some cases, resulting in prolonged pushing and operative vaginal births due to slow progress. If allowing 2 h (or 3 h with epidural) before pushing, it might be possible to experience fewer cases of dystocia and have fewer augmentation and possibly fewer operative vaginal births.

Uterine hyper-stimulation might partly explain the increased risk of operative vaginal births. Bernitz et al. found an OR at 3.7 of operative vaginal births and an OR at 2.5 of episiotomies in first-time mothers without dystocia being augmented with oxytocin compared with first-time mothers without dystocia not being augmented [1]. A Swedish multicenter study on a mixed population of parities showed an almost four-fold risk of operative vaginal births when women without dystocia were augmented with oxytocin compared with women without dystocia not being augmented [12].

A randomized controlled trial on 630 Swedish first-time mothers at term compared oxytocin after 2 h with oxytocin after 5 h without progress in the first stage of labor (4-9 cm). No differences were noted in mode of delivery and neonatal or maternal outcomes, except for the duration of labor, which was increased by 85 min [31]. A Cochrane review from 2013 comparing delayed versus early oxytocin in cases of slow progress of labor did not find any differences in mode of delivery and maternal and fetal outcomes, except for labor duration, which increased by 2 h [3]. Thus, one might assume that it is safe to use guidelines that allow a longer period of conservative management before augmentation with oxytocin than the guidelines used in our study.

## Conclusion

Our study shows that an intervention can reduce the use of oxytocin without increasing the rate of complications in labor. Even though the intervention was efficient, a substantial proportion of women received oxytocin without indication after the intervention. In our population, augmenting labor with oxytocin seemed to be associated with operative vaginal birth. The optimal guidelines for dystocia and augmentation may be even less strict than they were in this study, allowing labor to progress more slowly, especially early in the first stage and in the second stage of labor.

## Abbreviations

BMI: Body Mass Index
CS: Caesarean section
OVD: Operative vaginal delivery
PVO: The personal data officer
REC: Regional committee for health and research ethics
SPSS: Statistical package for social sciences
SVD: Spontaneous delivery
